# An overview of the healthy aging program for PinoY (HAPPY) senior citizens research: A cross-sectional study among community-dwelling older Filipinos

**DOI:** 10.3934/publichealth.2025029

**Published:** 2025-05-09

**Authors:** Robby Carlo A. Tan, Kyler Kenn M. Castilla, Angely P. Garcia, Kristine D. Macatangay, Shelley Ann F. de la Vega, Michael E. Serafico, Marco Mensink, Lisette de Groot

**Affiliations:** 1 Department of Science and Technology, Food and Nutrition Research Institute, Taguig City, Metro Manila, Philippines; 2 Division of Human Nutrition & Health, Wageningen University & Research, Wageningen, the Netherlands; 3 Institute on Aging, National Institutes of Health, University of the Philippines Manila, Manila City, Metro Manila, Philippines

**Keywords:** aging, nutrition, health, older adults, community-dwelling

## Abstract

**Background:**

As an effort in aligning with the United Nations Decade of Healthy Ageing, this paper reflects the methods and population characteristics from a comprehensive health and nutrition study among older Filipino adults.

**Methods:**

A cross-sectional study was conducted among older adults aged ≥60 years (*n* = 562) across the country. Descriptive statistics, chi-square, and *t*-tests were performed by sex and age.

**Results:**

The overall mean age was (69.4 ± 6.3) years. The Body Mass Index was significantly higher among those 60–69 years old compared to those >70. The lifestyle and health information varied in terms of significance between the sex and age groups.

**Discussion:**

This comprehensive study presented the profile of participants and a detailed methodology. Utilizing the data is highly encouraged to develop evidence-based policies relevant to the country driven by the demographic shift.

## 1. Introduction

Aging of the population is an inevitable phenomenon experienced by all nations, and it is estimated that by 2030, one out of six people will be 65 years old and over [1]. In the Philippines, adults aged 60 years and older represented about 6% of the country's total population in the year 2000, and within two decades later, it increased to about 8.5% [2],[3]. The demographic shift to an aging population entails an increased demand for supportive health care and social systems [4],[5].

Older adults are at an increased risk for malnutrition due to factors inherent to aging such as a decreased appetite, loss or changes in taste and smell, fatigue, dysphagia, and other coexisting morbidities [6]. A decreased functional capacity, muscle loss, anemia, a decreased immunity, a reduced cognitive function, a higher risk of infection, poor wound healing, and the development of chronic non-communicable diseases are consequences associated with malnutrition [7]–[9]. These contribute to an increased morbidity and mortality among the older adult population [10],[11].

Malnutrition is one of the geriatric syndromes detected through a comprehensive geriatric assessment (CGA) [12]. CGA is a multidisciplinary diagnostic and management process in which the multiple problems and needs of an older adult are identified, and a coordinated care plan is developed [13]. In the Philippines, the Focused Interventions for Frail Older Adults Research and Development Project (FITforFrail) is one of the first few research studies in the country that utilized CGA in the community setting [14].

In the past, studies conducted among older Filipinos were performed in hospitals and institutional settings [15]. In a university setting, Laude et al. reported on the nutrition and frailty status of working and retired employees of University of the Philippines Los Baños (UPLB) [16]. Another local study investigated the quality of life, health status, health behavior, household relations, and community participation among community dwellers in Iloilo City [17]. On a national level, the National Nutrition Survey (NNS) of the Department of Science and Technology - Food and Nutrition Research Institute (DOST-FNRI) has been conducted every five years since it commenced in 1978. Data across all age groups on nutrition and health indicators, including anthropometric, biochemical, clinical and health, and dietary intakes among others, are collected and disseminated to the public [18]. In 2018, the Longitudinal Study of Ageing and Health in the Philippines (LSAHP) was implemented wherein data on the health, economic status, and well-being from a nationally representative sample of Filipinos aged 60 years and above were reported [19]. Despite all these relevant local studies conducted, data gaps on nutrition research exist. In particular, there is a need to understand the interrelationship of various factors surrounding the nutritional status of older Filipinos such as functional capacity, body composition, other health outcomes, environmental factors, biological indicators, and their quality of life. Hence, the Healthy Aging Program for Pinoy (HAPPY) Senior Citizens research study was implemented in the Philippines, which is aimed at generating comprehensive data that would shed light on the nutrition, health, and overall well-being of older Filipinos.

This paper aims to describe the sociodemographic, anthropometric measurements, lifestyle, and health information profile of the participants by sex and age. In addition, the overall methodologies of the HAPPY Senior Citizens research study are presented. Being a low-and-middle income country with a relatively young population, this research study is an important milestone in the Philippines Nutrition and Health research scene in aligning its efforts with the thrust of the United Nations for Decade of Healthy Ageing [20], which pushes to generate science-based evidences to proactively create programs and policies that would respond to the inevitable demographic shift in the coming years. Other countries with similar demographic structures and economic situations can also benefit from this research study.

## 2. Materials and methods

### 2.1. Study site and collection period

**Figure 1.**
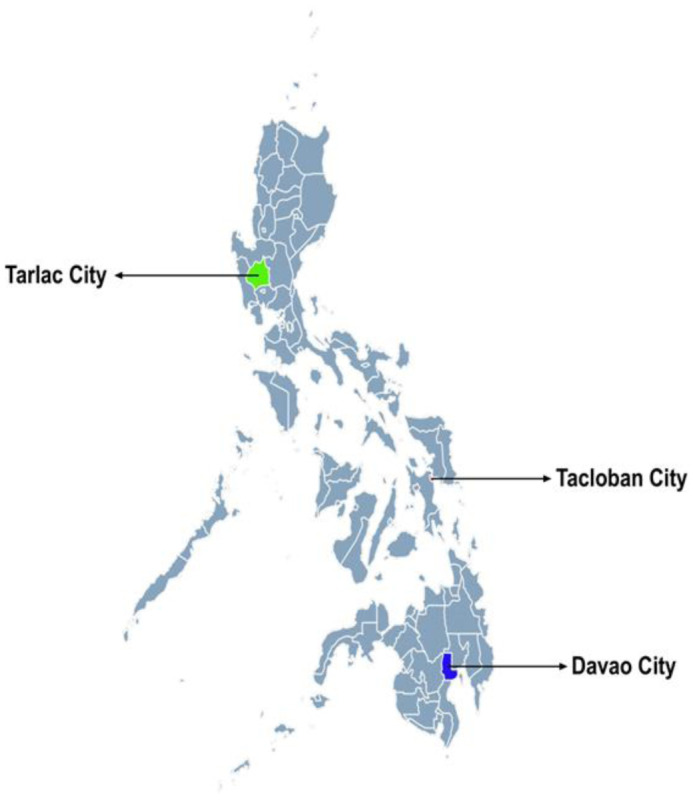
Location of study sites.

From November 2021 to June 2022 amidst the COVID-19 pandemic in the Philippines, a cross-sectional study was conducted among Filipino community-dwelling older adults aged 60 years and above from one city of each of the three major island groups in the Philippines, namely Tarlac City for Luzon, Tacloban City for Visayas, and Davao City for Mindanao (Figure 1). The motivation of selecting one city from each of the major island groups was to have a snapshot of the older Filipinos' nutrition and health situation at that period. A descriptive overview of each city is presented in Table 1.

**Table 1.** Overview of the selected study sites from each major island group in the Philippines.

**Cities**	Ref	Tarlac	Tacloban	Davao
**Island group**		Luzon	Visayas	Mindanao
**Province / Region**		Tarlac / Region III	Leyte / Region XIII	Davao Del Sur / Region XI
**Land area *(square kilometers)***	[21]	274.66	201.72	2443.61
**Geographic location**		Landlocked	Coastal	Coastal
**Urbanization classification**	[22]–[24]	Urbanized City	Highly Urbanized City	Highly Urbanized City
**Total population**	[25]	342,493	242,089	1,632,991
**Total population of senior citizen**	[25]	25,714	16,269	109,562

### 2.2. Sampling design and participant selection

Working around the restrictions imposed during the collection period, the three cities were purposely selected based on the following criteria: 1) presence or proximity of available CGA-trained geriatricians or physicians; 2) total population of the city vis-a-vis the population of senior citizens; 3) safety concerns; 4) accessibility; and 5) feasibility of conducting the research during the period.

The local government units with jurisdiction over the cities of Tarlac, Tacloban and Davao coordinated and accordingly requested to identify 10 potential *barangays* (brgy.), which is the smallest unit of administrative division in the country, for each city to be included in the study. From the ten barangays, four were randomly selected to take part in the study.

A list of residents aged 60 years and above for each *barangay* was requested through the assistance of the Office of the Senior Citizens Affairs. From the list, 200 older adults were randomly selected for each city from the Philippine Statistics Authority (PSA) sampling frame. Then, the list of selected participants was endorsed to the respective *barangays* for coordination, and the verification was performed by the *barangay* health workers. Randomly drawn participants who refused or could not be located at the time of coordination and verification (e.g., deceased, changed address, unavailability due to health reasons) were conveniently replaced by another participant within the same *barangay* but were matched in terms of a similar age range (60–69 or 70 and above) and sex.

Face-to-face recruitment of the pre-coordinated study participants was performed by the research team with the help of the *barangay* health and nutrition workers. The household locations of each participant were visited by the researchers to verify the eligibility to participate based on the inclusion and exclusion criteria of the study. The inclusion criteria for the study participants were as follows: 1) community-dwelling older adults aged 60 years and above; 2) ability to comprehend and provide consent to join the study; 3) ability to answer questions on health, nutrition, and other relevant information; and 4) ambulatory and can stand alone. The excluded participants were those who met the following criteria: 1) bedridden; 2) unable to comprehend and answer interview questions; and 3) has a severe illness/condition (e.g. cancer, transplant).

The non-eligible participants were replaced with other older adults who resided within the study site based on recommendations of the health and nutrition workers. The research was complied with the existing COVID-19 protocol of each city and *barangay* during the time of collection.

The project received an ethical clearance from the Food and Nutrition Research Institute Institutional Ethics Review Committee (FIERC No. 2021–017). Signed informed consent was sought from each participant after thorough explanation of the project. The study was conducted in accordance with the Declaration of Helsinki.

### 2.3. Sample size calculation

One of the objectives of the HAPPY Senior Citizen research study is to collect information on the body composition and functional capacity of older persons. Sarcopenia is one of the emerging interests in aging, which is defined as the age-related loss of muscle mass, muscle strength, and physical performance [26]. The G*Power software version 3.1 was used to calculate the sample size by city using a priori analysis to determine the minimum sample size based on the 22.0% prevalence of sarcopenia among Malaysian community-dwellers aged 60 years and above [26], with 95% confidence level and a 5% margin of error [27]. A minimum sample size of 264 for the entire study was required to derive relevant implications on the research topic. A maximum of 600 participants was targeted for the entire study, which accounted for a 30% drop out rate and the absence of complete randomization at the site selection.

### 2.4. Variables collected

#### 2.4.1. Sociodemographic information

A researcher-administered questionnaire was utilized to gather data on the participants age, birthplace, sex, civil status, household size, length of stay in the household, educational attainment, financial resources, occupational history, toilet type, source of drinking water, mode of food preparation, and health care arrangements.

#### 2.4.2. Anthropometry and body composition

The body circumferences were measured using a retractable, non-stretchable tape (Seca® 201, Hamburg, DE). The waist circumference was measured at the midpoint between the lowest rib and the iliac crest. The hip and calf circumferences were measured at the point of largest circumference over the buttocks and calf, respectively [27],[28]. The mid-upper arm circumference was taken at the midpoint between the acromion and the olecranon process [18]. The circumferences were taken such that the tape was firmly wrapped around the point of measurement yet not squeezing the skin.

The knee height was obtained using a large caliper (Seca® 207, Hamburg, DE) in a sitting position and the leg bent at a 90° angle. The demi-span was measured in a standing erect position with one arm stretched parallel to the shoulder [29]. The height was measured using a portable stadiometer (Seca® 213, Hamburg, DE) without any footwear and headwear. The weight and body composition were collected using a multi-frequency segmental body impedance analyzer (BIA) (Tanita® MC-780MA, Tokyo, JP) following the manufacturer's protocol. The nutritional status by the Body Mass Index (BMI) was determined using the World Health Organization (WHO) BMI classification [18].

#### 2.4.3. Biological samples

The participants were informed to observe 10–12 hours of fasting and were requested not to drink alcoholic beverages nor smoke 24 hours prior to the day of collection. A trained, licensed medical technologist extracted 8 cc to 10 cc of blood via venipuncture. Urine specimens were collected early in the morning and stored in sample containers for a urinary iodine analysis. Blood analyses (hemoglobin, lipid profile, c-reactive protein, and glycosylated hemoglobin) and the Urinary Iodine Excretion analysis were performed at the DOST-FNRI SLG following standard laboratory protocols.

With the growing interest in microbiome studies in aging, a pea-size stool sample was collected from a randomly selected subsample of 20% of the total participant in each city.

#### 2.4.4. Dietary intake

A trained registered nutritionist-dietitian administered the 110- item food frequency questionnaire using the information from the previous three months as the reference period to determine the habitual intake. Food items were identified based on the commonly consumed food groups from the NNS database [30],[31].

#### 2.4.5. Physical activity level and lifestyle

The WHO Global Physical Activity Questionnaire was used to capture information on physical activity [32]. The 16-item questionnaire collects data on four domains: at work, during travel to and from places, recreational activities, and one sedentary behavior (sitting). The frequency per week (days), the duration (minutes) spent performing different activities by intensity (moderate and vigorous), and the average time spent sitting in a day were asked.

#### 2.4.6. Functional capacity and physical performance

Functional capacity through grip strength was assessed using a hand-held dynamometry (Jamar®, IL, US). The Timed Up-and-Go (TUG) test and the Short Physical Performance Battery (SPPB) test were used to assess physical performance, which measures functional mobility and overall lower extremity function among older people, respectively [33]–[35].

For the 6-minute walk test, the completion time of the participants was assessed by walking a 20-meter pathway twenty times in a comfortable manner. The 6-Minute Walk Test was performed to measure the aerobic capacity and endurance.

#### 2.4.7. Sarcopenia and frailty screening

The SARC-F questionnaire is a screening tool that screens patients for self-reported signs suggestive of sarcopenia [36]. It is composed of five questions which include deficiencies in the following: 1) strength; 2) assistance in walking; 3) rise from a chair; 4) climb stairs; and 5) falls. Each self-reported parameter receives a minimum and maximum score of 0 and 2, respectively, with the greatest maximum SARC-F score being 10.

The Frailty Index was assessed using Fried's frailty criteria, specifically shrinking, physical endurance/energy, low physical activity, weakness, and slow walking speed. Each criterion has a frailty cut point which determines the overall frailty status of the participant. Based on the scores, the participants can be divided into three stages: non-frail or robust (score 0), pre-frail (score 1–2), and frail (score 3–5) [37].

#### 2.4.8. Comprehensive geriatric assessment

The comprehensive geriatric assessment is a multidimensional diagnostic process which aims to derive a holistic approach to maximizing the overall health of the aging population. The Filipino version of the form used in this research [38], which is a questionnaire-based assessment used to assess the older person, was translated and validated by the Institute on Aging of the National Institutes of Health, University of the Philippines Manila, Philippine College of Geriatric Medicine, and Department of Health.

After the interview, the CGA-trained geriatrician or physician reviewed the responses before proceeding to the physical and mental examination and diagnosis.

#### 2.4.9. Quality of life

The perception of their overall quality of life was assessed using the WHO Quality of Life Brief Version (WHOQOL-BREF), which has been culturally validated for Filipino older adults [39],[40]. The 26-item questionnaire covers four domains of life: physical health, psychological, social relationships, and environment. Each item is scored through a 5-point ordinal scale which is dependent on the participant's degree of perceived capability, frequency, or contentment on specific facets of their life within the last two weeks. An additional question on the perceived challenges during the time of COVID-19 was added to gather their insights and experiences.

#### 2.4.10. Geographical information system

The locations of households and landmarks of each *barangay* were recorded using the Global Positioning System (GPS) from smartphones (Save Location app). These GPS points were verified and mapped in Google Earth together with the environmental data. Additionally, the environmental data related to the neighborhood greenness, urbanization, population, food proximity, and topography were identified. Using a geospatial software, the nutrition and health outcomes were mapped at the neighborhood levels to provide spatial information on the health and nutritional status.

Further details of the methodology are reflected in Supplementary File 1.

### 2.5. Data management and statistical analysis

The collected information was encoded, reviewed, and counter checked before storing in a secured database location. The data from the paper-based forms were encoded by trained research assistants using EpiInfo™, Version 7.2.4.0. A coding manual was prepared and utilized in the data management. The encoded data was counterchecked and sent to an independent statistician/data check for random validation. All analyses were performed using IBM SPSS® (version 23, IBM, Armonk, NY, USA). The results with *p* values of < 0.05 were considered as statistically significant.

The continuous data were checked for normality distribution through visual plots and the Shapiro-Wilk Test. The descriptive data were reported as either mean and standard deviation or median and interquartile range, while the categorical data were expressed in count and percentages. The data were analyzed by sex and age group of 60–69 and 70 and above years old. The difference between groups was determined using the Student *t*-test for equal variances and Welch's *t*-test for unequal variances. The Chi-square test was used for categorical variables. With the wealth of information gathered in this study, a future use of the database for research and policy making purposes is highly encouraged. Table 2 presents the different variables gathered and the details of the outcome measures. The datasets generated and/or analyzed are available from the corresponding author upon reasonable request and with approval from DOST-FNRI and the principal investigator.

**Table 2.** Summary of variables collected and details of the outcome measures.

Component	Variables/ Details
1. Sociodemographic	Age Sex Civil status Household size and dependency Financial source Educational attainment Length of stay Religion Employment status Water source Food preparation
2. Anthropometry and Body Composition	Height, Weight, Body Mass Index Body circumferences (waist, hip, mid-upper arm, calf) Knee height Demi-span Body Fat Fat Free Mass Muscle Mass Total Body water
3.Biological Samples (Blood, Urine, Stool)	Hemoglobin Glycosylated hemoglobin (HbA1c) Lipid profile C-reactive protein Urinary iodine excretion Nutrigenomics/Genomics* Microbiome*
4. Diet	Food Frequency Questionnaire
5. Lifestyle	Physical Activity Level Smoking Alcohol Consumption
6. Functional Capacity and ‘Physical Performance	Hand Grip Strength Short Physical Performance Battery Timed Up and Go 6-minute walk test
7. Sarcopenia and Frailty Screening	Fried Frailty Index SARC-F
8. Comprehensive Geriatric Assessment	Problems with senses Sleeping patterns/problems Fall history and walking problems Medical history - illnesses, surgery, medications Nutrition - supplements Immunization history Activities of Daily Living Review of systems Physical and mental health status Vital Signs Health Insurance Status Health Consultation COVID-19 vaccination status
9. Quality of Life	Physical Health Domain Psychological Domain Environmental Domain Social Domain Overall Quality of Life
10. Geographic Information System	Global Positioning System

Note: *Stored biological samples.

## 3. Results

**Figure 2.**
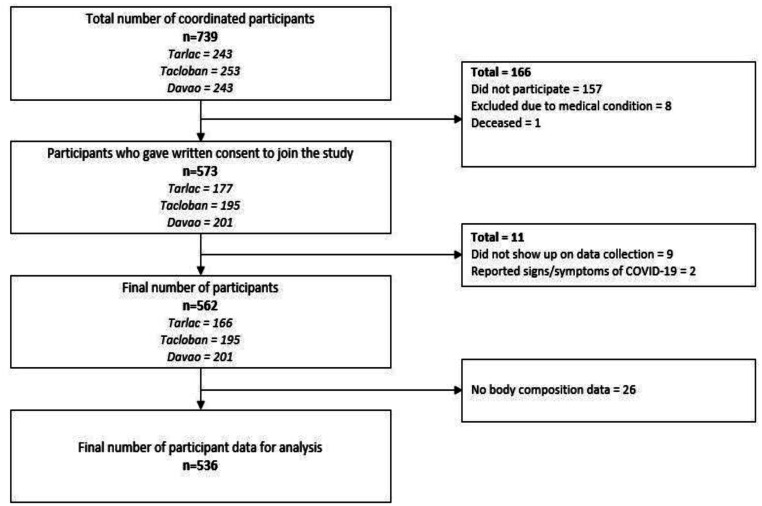
Participant screening and recruitment.

Table 3 reflects the total population of older adults in the *barangay* based on the 2015 census [25] and the actual number of study participants by city and *barangay*, while the overall recruitment process that led to a total of 562 older adult participants for the study is depicted in Figure 2.

**Table 3.** The 2015 Philippine Statistics Authority (PSA) Population Census of older adults aged 60 years old above and the actual number of study participants, by *barangay*.

**Study site (City and *barangay*)**	**60 and above (2015, PSA) [25]**	**Actual number of study participants**
Tarlac City	**25,714**	**166**
*Brgy*. Mapalacsiao	486	22
*Brgy*. San Isidro	776	36
*Brgy*. San Rafael	1274	62
*Brgy*. Tibag	1086	46
Tacloban City	**16,269**	**195**
*Brgy*. 6	53	41
*Brgy*. 92	311	34
*Brgy*. 96	466	41
*Brgy*. 99	313	79
Davao City	**109,562**	**201**
*Brgy*. 19B	2214	98
*Brgy*. 5A	530	31
*Brgy*. Acacia	216	14
*Brgy*. Mandug	1264	58

The overall mean age was 69.4 (6.3) years, and no significant differences were observed for the mean age across the three study sites. Overall, more than half of the participants belonged to the 60–69 age group (57.1%), with more females being included (61.0%, *p* < 0.01) than males, as shown in Table 4.

**Table 4.** Age and sex distribution of the study participants by city.

**Project**	**Overall (*n* = 562)**	**City**			***p*-value**
		**Tarlac (*n* = 166)**	**Tacloban (*n* = 195)**	**Davao (*n* = 201)**	
Age, years	69.4 (6.3)	69.4 (5.95)	69.7 (7.0)	69.2 (5.9)	0.71
Age group					0.96
60–69	321 (57.1)	94 (56.6)	113 (57.9)	114 (56.7)	
70 above	241 (42.9)	72 (43.4)	82 (42.1)	87 (43.3)	
Sex					**<0.01**
Male	219 (39.0)	57 (34.3)	65 (33.3)	97 (48.3)	
Female	343 (61.0)	109 (65.7)	130 (66.7)	104 (51.7)	

*Note: Values presented are mean (SD). Significant value (p < 0.05) is written in bold*.

Table 5 presents the sociodemographic profile of the participants by sex and age groups. Most of the participants were married (49.8%), attained an elementary level of education (42.3%), and were members of the senior citizen's organization (84.7%). Most of their financial sources came from their families (66.4%) and/or pension (48.8%). A significant difference was observed between their educational attainment and the age group classification, with a lower educational level in the older age group. Those belonging to the younger age subgroup attained higher levels of education. In addition, in terms of the presence of caregiver, a difference was present between the 60–69 years old and the 70 and above age groups, with the latter group having more caregivers (10.9% *vs*. 2.5%, *p* < 0.01). No significant differences were found in the number of household members and the social group participation for both sex and age groups.

The nutritional status based on BMI is reflected in Table 6: about half (46.5%) had a normal BMI, while overweight and obese individuals had a combined prevalence of 42.4%. The mean BMI was significantly higher among the 60–69 age group than the 70 and above age group. In terms of sex, females have significantly higher BMIs (24.4 *vs*. 23.5; *p* = 0.02) compared to males. There were significantly more male smokers (25.6% *vs*. 5.5%, *p* < 0.01) and alcohol drinkers (50.2% *vs*. 26.5%, *p* < 0.01) compared to females. A significant difference between the 60–69 years old and the 70 and above age groups was only observed in terms of alcohol consumption, with a higher intake in the younger group.

**Table 5.** Sociodemographic profile by sex and age group.

**Project**	**Overall**	**Sex**		***p*-value**	**Age group**		***p*-value**
		**Male**	**Female**		**60–69**	**70 above**	
Number of household members (%)							
1–3	212 (37.9)	76 (34.7)	136 (39.9)	0.43	107 (33.5)	105 (43.6)	0.05
4–6	200 (35.7)	84 (38.4)	116 (34.0)		123 (38.6)	77 (32.0)	
7 above	148 (26.4)	59 (26.9)	89 (26.1)		89 (27.9)	59 (24.5)	
Educational attainment (%)							
Elementary	236 (42.3)	89 (40.6)	147 (43.4)	0.76	102 (32.1)	134 (55.8)	**<0.01**
Secondary	193 (34.6)	77 (35.2)	116 (34.2)		125 (39.3)	68 (28.3)	
Tertiary	129 (23.1)	53 (24.2)	75 (22.1)		91 (28.6)	38 (15.8)	
Civil status (%)							
Single	37 (6.6)	11 (5.0)	26 (7.6)	**<0.01**	23 (7.2)	14 (5.8)	**<0.01**
Married	280 (49.8)	155 (70.8)	125 (36.4)		180 (56.1)	100 (41.5)	
Widowed	214 (38.1)	41 (18.7)	173 (50.4)		98 (30.5)	116 (48.1)	
Separated/Divorced	31 (5.5)	12 (5.5)	19 (5.5)		20 (6.2)	11 (4.6)	
Financial source (%)							
Salary	77 (13.7)	40 (18.3)	37 (10.8)	**<0.01**	62 (19.3)	15 (6.2)	**<0.01**
Pension	274 (48.85)	111 (50.7)	163 (47.5)	0.46	130 (40.5)	144 (59.8)	**<0.01**
Family	373 (66.4)	135 (61.6)	238 (69.4)	0.06	203 (63.2)	170 (70.5)	0.07
Business	131 (23.3)	51 (23.3)	80 (23.3)	0.99	79 (24.6)	52 (21.6)	0.40
Others	5 (0.9)	2 (0.9)	3 (0.9)	0.96	4 (1.2)	1 (0.4)	0.30
Presence of caregiver (%)	34 (6.1)	16 (7.4)	18 (5.3)	0.31	8 (2.5)	26 (10.9)	**<0.01**
Social group participation (%)							
Senior Citizen's Org	476 (84.7)	186 (84.9)	290 (84.5)	0.90	271 (84.4)	205 (85.1)	0.83
Church	127 (22.6)	41 (18.7)	86 (25.1)	0.08	74 (23.1)	53 (22.0)	0.77
Alumni	18 (3.2)	7 (3.2)	11 (3.2)	0.99	11 (3.4)	7 (2.9)	0.73
Volunteer	31 (5.5)	17 (7.8)	14 (4.1)	0.06	107 (5.3)	14 (5.8)	0.79
Others	8 (1.4)	6 (2.8)	2 (0.6)	0.21	5 (1.6)	2 (0.8)	0.44
City (%)							
Tarlac	166 (29.5)	57 (26.0)	109 (31.8)	**<0.01**	94 (29.3)	72 (29.9)	0.96
Tacloban	195 (34.7)	65 (29.7)	130 (37.9)		113 (35.2)	82 (34.0)	
Davao	201 (35.8)	97 (44.3)	104 (30.3)		114 (35.5)	87 (36.1)	

Note: Values presented are *n* (%). Significant values (*p* < 0.05) are written in bold. Missing values: No. of household members = 2, Educational attainment = 4, Presence of caregiver = 5.

No significant differences were observed between the sex and age groups in regards to other health information (self-perceived ability to support daily financial and healthcare needs, availing of health insurance, consulting with healthcare providers, COVID-19 vaccination, and history of falls).

The blood pressure level (*p* = 0.02) and hemoglobin status (*p* < 0.01) were significantly different between the younger and older age subgroups, with a higher blood pressure and a lower hemoglobin status in the older age group. No differences were found between both sexes. However, with regards to the total cholesterol, females had a significantly higher combined prevalence of high and borderline high cholesterol (53.9%, *p* < 0.01) than their male counterparts. No significant differences in the cholesterol level and status between the age groups were seen.

**Table 6.** Nutritional Status, Lifestyle, and Health Information of the study participants, by sex and age group.

**Project**	**Overall**	**Sex**		***p*-value**	**Age group**		***p*-value**
		**Male**	**Female**		**60–69**	**70 above**	
Body Mass Index (kg/m^2^)							
Underweight (*n*, %)	61 (11.1)	27 (12.7)	34 (10.1)		26 (8.3)	35 (14.9)	
Normal (*n*, %)	265 (46.5)	107 (50.5)	149 (44.1)	0.05	129 (41.0)	127 (54.0)	**<0.01**
Overweight (*n*, %)	187 (34.0)	68 (32.1)	119 (35.2)		126 (40.0)	61 (26.0)	
Obese (*n*, %)	46 (8.4)	10 (4.7)	36 (10.7)		34 (10.8)	12 (5.1)	
Current smokers (*n*, %)	75 (13.3)	56 (25.6)	19 (5.5)	**<0.01**	51 (15.9)	24 (10.0)	0.12
Current alcohol drinkers (*n*, %)	201 (35.8)	110 (50.2)	91 (26.5)	**<0.01**	137 (42.7)	64 (26.2)	**<0.01**
Perceived ability to support daily expenses (*n*, %)	229 (40.7)	88 (40.2)	141 (41.1)	0.83	137 (42.7)	92 (38.2)	0.28
Perceived ability to support healthcare needs (*n*, %)	404 (72.5)	154 (71.0)	250 (73.5)	0.51	233 (73.5)	171 (71.2)	0.55
Availment of health insurance (*n*, %)	405 (72.5)	161 (73.9)	244 (71.6)	0.55	227 (70.9)	178 (74.5)	0.35
Ever consulted with healthcare provider (*n*, %)	406 (72.4)	151 (68.9)	255 (74.6)	0.15	230 (71.9)	176 (73.0)	0.76
COVID-19 vaccine status (*n*, %)							
Complete	413 (73.5)	160 (73.1)	253 (73.8)	0.85	246 (76.6)	167 (69.3)	0.15
Incomplete	20 (3.6)	9 (4.1)	11 (3.2)		10 (3.1)	10 (4.1)	
Unvaccinated	129 (23.0)	50 (22.8)	79 (23.0)		65 (20.2)	64 (26.6)	
Medication intake (*n*, %)							
No medication	223 (39.7)	94 (42.9)	129 (37.6)	0.45	129 (40.2)	94 (39.0)	0.81
1–2	243 (43.2)	90 (41.1)	153 (44.6)		140 (43.6)	103 (42.7)	
3 above	96 (17.1)	35 (16.0)	61 (17.8)		52 (16.2)	44 (18.3)	
History of fall for the past 3 months (*n*, %)	72 (12.8)	22 (10.0)	50 (14.6)	0.11	38 (11.8)	34 (14.2)	0.46
Blood pressure (*n*, %)							
Normal	62 (11.3)	25 (11.8)	37 (10.9)	0.97	35 (11.1)	27 (11.5)	**<0.02**
Prehypertension	216 (39.3)	84 (39.6)	132 (39.1)		137 (43.5)	79 (33.6)	
Hypertension stage 1	138 (25.1)	51 (24.1)	87 (25.7)		81 (25.7)	57 (24.3)	
Hypertension stage 2	134 (24.4)	52 (24.5)	82 (24.3)		62 (19.7)	72 (30.6)	
HbA1c status (*n*, %)							
Normal	237 (43.9)	94 (44.5)	143 (43.5)	0.76	125 (40.3)	112 (48.7)	0.11
Prediabetes	192 (35.6)	77 (36.5)	115 (35.0)		114 (36.8)	78 (33.9)	
Diabetes	111 (20.6)	40 (19.0)	71 (21.6)		71 (22.9)	40 (17.4)	
Hemoglobin status (*n*, %)							
Normal	482 (89.1)	190 (90.0)	292 (88.5)	0.57	292 (93.9)	190 (82.6)	**<0.01**
Low	59 (10.9)	21 (10.0)	38 (11.5)		19 (6.1)	40 (17.4)	
Total Cholesterol status (*n*, %)							
Desirable	248 (46.1)	113 (54.1)	135 (41.0)	**<0.01**	141 (45.8)	107 (46.5)	0.68
Borderline high	178 (33.1)	67 (32.1)	111 (33.7)		99 (32.1)	79 (34.3)	
High	112 (20.8)	19 (13.9)	83 (25.2)		68 (22.1)	44 (19.1)	

Note: Values presented count (percentage). Significant values (*p* < 0.05) are written in bold. Missing values: Physical Activity = 43, Availment of health insurance = 3, Ever consulted with healthcare provider = 1, History of fall for the past 3 months = 1, Blood pressure = 1.

## 4. Discussion and recommendation

### 4.1. Discussion

The HAPPY Senior Citizens, a cross-sectional study, was conducted to generate comprehensive data on the nutrition, health, functional status, and quality of life of community-dwelling older adults in three provincial cities in the Philippines covering a total of 12 *barangays*. A total of 562 older adults participated in the study. This paper was able to generate the profile of the study participants in terms of their sociodemographics, nutritional status, lifestyle, and general health characteristics. Furthermore, the overall methodology of the HAPPY Senior Citizens research study was presented in the detail with the following 10 components: 1) Sociodemographic; 2) Anthropometry and Body Composition; 3) Biological Samples; 4) Diet; 5) Lifestyle; 6) Functional Capacity and Physical Performance; 7) Sarcopenia and Frailty Screening; 8) CGA; 9) Quality of Life; and 10) GIS. The comprehensive nature of this study presents an opportunity to understand the interrelationships of factors in nutrition, health, and aging research in the country and the region.

The data presented in this paper provide a reasonably good representation of the older Filipino population as their characteristics aligned with previous databases namely the PSA [25], NNS, Longitudinal Study on Ageing and Health (LSAHP), and the Focused Interventions for Frail Older Adults Research project (FITforFrail). Specifically, more older adults were females, belonged to the younger old subgroup, and were married. The HAPPY Senior Citizens project complements these studies with the added detailed information on nutrition, functionality, health, and quality of life using comprehensive assessment tools and instruments.

In the Philippines, the NNS is one of the largest cross-sectional health surveys conducted in the country since 1978. The NNS covers various age and physiologic groups from nationally represented samples across the country. Since 2013, data collected in the NNS concerning the older adults included anthropometry, biochemical, clinical markers, dietary intake, lifestyle, sociodemographic, and government participation, with diet as the primary exposure variable [18]. However, age-sensitive parameters such as body composition and functional status were not included in the collection. These parameters are key indicators that influence healthy aging [1],[41],[42]. The HAPPY Senior Citizens study is able to fill this gap with a representative sample of community-dwelling older adults from three provincial cities in the Philippines, thereby including body composition and functional measurements.

Two other initiatives in the Philippines align with our present study: LSAHP and the FITforFrail project. The LSAHP was conducted from October 2018 to February 2019 to investigate the health status and its determinants among community-dwelling older adults across the country. A total of 5985 older adults participated in the baseline survey, which collected data on health, economic status, and overall well-being. The LSAHP collected information on body composition and functional status. However, dietary intake was not included in their reported methodology. A poor diet has been identified to be one of the major modifiable risk factors for chronic disease [43]. Thus, an assessment of dietary intake is crucial to determine its role in fostering a healthy aging process.

The FITforFrail project was conducted in select regions in the country between 2017 and 2020, and provided a snapshot of how the health system fares to the WHO healthy ageing framework alongside describing the health status of community-dwelling older adults in 4 regions. In terms of the findings related to nutrition, the study found that 30.3% were at risk for malnutrition and 4.4% were malnourished. Among those identified to be malnourished, 77.9 % reported an inadequacy of finances and 94.4% claimed that they were worried about their ability to support their healthcare needs [14].

The NNS, LSAHP, and FITforFrail are commendable large-scale studies in the country that collected vital information from older adults; however, this current study complemented these earlier researches by bridging the key variables to complete the picture of healthy aging in the context of nutrition research in the country. The results of the Happy Senior Citizens study complemented the methodologies used in previous studies and contributed to the available local and regional data.

However, this study was conducted during COVID-19 pandemic in the Philippines, wherein strict health and safety public health measures were imposed. As one of the vulnerable groups, older adults were discouraged or not allowed to leave their home [44]. A difficulty in the recruitment of older adults was a concern because of the hesitation to join, thereby citing health risks as a reason. The participants who showed COVID-19 symptoms at any point of the study were immediately excluded from participation to safeguard the health of both the researchers and participants. This led to the reduction of the total number of two (2) participants.

The percent distribution of this study in terms of sex, with 39.0% for males and 61.0% for females, and age, with 61.7% for 60–69 years and 38.3% for 70 years and above, was comparable to the distribution reported in the 2018 NNS, with 41.7% for males and 58.3% for females in terms of sex, and 57.1% and 42.9% for 60–69 years and 70 years and above, respectively, in terms of age. More than half of the participants belonged to the young old age sub-group (60–69) and were females. This also affirms the similarity between the 2018 NNS and our study considering the limitations of our study. This can be attributed to the overall proportion of female older adults in the country as well as the longer life expectancy of females [14],[45].

Furthermore, most of the participants in this study were married and were in consonance with the proportion of married senior citizens in the Philippines [25]. The findings of this paper relevant to educational attainment, membership with senior citizens organizations, and living arrangements were also consistent with the LSAHP [45] and FITforFrail [14]. Most older adults live with others, attained elementary school as their highest level of education, and are members of senior citizens organizations.

The findings from this study revealed that more than half of the participants relied on their families for financial support which highlights the financial insecurity among older Filipino adults. This is consistent with the findings of FITforFrail, where 77.3% mostly received financial support from their children [14]. According to a study by Carandang et al., older adults experienced difficulties saving for retirement due to the high prevalence of an informal workforce in the country, and a low salary resulted in the prioritization of their own family's financial needs over theirs. Due to this financial burden combined with the Filipino culture of family members taking care of the older adults, an increased reliance on intergenerational family support is prevalent among the aging population [46].

In terms of nutritional status, similar trends were observed among the sex and age groups between the current study and the NNS. Males and those aged 70 and above had a higher prevalence of being underweight. Meanwhile, females and those aged 60–69 years old had a higher prevalence of being either overweight or obese [18]. This agrees with a systematic review conducted among the older population which reported that older women have a larger fat mass than men, while older men have a greater skeletal muscle mass than women [47].

In terms of lifestyle-related risk factors for non-communicable diseases, there were more male smokers and alcohol drinkers than females in this study. A possible explanation to this observation is that females are less likely to engage in risky health behaviors such as smoking and alcohol consumption [48]. Furthermore, the age subgroup's alcohol consumption was found to be significant in this study and declined with age, which is consistent with the other studies among older adults in the country, namely Myanmar, Vietnam, and Thailand [49],[50].

### 4.2. Recommendation

The HAPPY Senior Citizens study provides valuable insights into the health and nutrition status of older Filipino adults, thereby offering a foundation to develop targeted interventions. Given the increasing proportion of older adults in the population, interventions should prioritize addressing malnutrition, improving functional capacity, and enhancing the overall quality of life through evidence-based policies and programs.

A key implication of this study is the need for comprehensive, age-specific nutrition and health programs tailored to the diverse needs of older adults. The high prevalence of overweight and obese individuals among younger seniors (60–69 years) and undernutrition among those aged 70 and above highlights the importance of customized interventions. Strategies such as nutrition education, community meal programs, and the promotion of balanced diets rich in protein and micronutrients can help mitigate these issues while encouraging healthy lifestyles.

Research has shown that age-related physiological changes and socioeconomic factors contribute to disparities in health outcomes, thus necessitating tailored public health interventions [51],[52]. Our findings reinforce the importance of nutrition-sensitive policies, physical activity promotion, and geriatric health assessments, particularly in resource-limited settings. By situating our results within this broader scientific context, we contribute to the ongoing discourse on aging and public health in low- and middle-income countries (LMICs).

Our findings hold relevance beyond the Philippines, as other LMICs undergoing similar demographic shifts face common challenges such as limited healthcare access, financial insecurity, and a growing burden of age-related diseases. The HAPPY Senior Citizens study serves as a model for collecting comprehensive health and nutrition data, thus offering a replicable framework to generate localized evidence in similar settings.

The dataset generated from this study provides a valuable resource for a further in-depth analysis, thus supporting the development of evidence-based policies, programs, and services tailored to the needs of older adults. By leveraging these findings, policymakers and researchers can craft targeted, data-driven strategies that promote healthy aging and ensure public health initiatives remain adaptive to the evolving needs of the aging populations.

## 5. Strengths and limitations

In the Philippines, there are limited nutrition and health studies that focus on older adults. In this study, the researchers collected different data such as sociodemographic, anthropometric measurements, body composition, biochemical and clinical markers, health information and lifestyle, functional capacity and physical performance, health status based on comprehensive geriatric assessment, and quality of life. In regard to this, a wide range of variables can be taken into account to address the issue of sarcopenia and other relevant health outcomes in the study population.

This study acknowledges the following limitations: 1) the study was conducted during the COVID-19 pandemic, hence the results should be interpreted under this context; 2) the selection of the study site was purposive; 3) originally, participants were randomly selected but replacements were identified through a convenience selection; 4) no causation can be derived in the study design; and 5) most of the data collected relied on memory of older adults. The COVID-19 pandemic may have affected the participation of the originally sample participants from the brgy. list. Hence, the replacements were invited through a convenience selection. To minimize potential bias, age and sex-matched participants were invited from the same barangay as replacements.

This study has several notable strengths. It utilized a multi-frequency segmental body impedance analyzer, thus ensuring a more precise body composition analysis on top of the BMI, alongside a comprehensive health assessment conducted by a CGA-trained physician. Additionally, it introduced a novel integration of GIS methods to evaluate the health and nutrition outcomes. The data collection was carried out by well-trained researchers using standardized procedures, thereby enhancing the reliability of the findings. The study encompassed one city from each of the three main island provinces in the country and increased the sample size to account for the sampling design. Since the study was conducted from November 2021 to June 2022, it provides valuable insights into the health and nutrition status of older Filipinos during the COVID-19 pandemic and the transition to the “new normal.”

Despite the limitations mentioned, this study stands as one of the most comprehensive investigations into the health and nutrition of the aging population in the country to date. Its findings have the potential to inform policies and practices, both locally and in regions with similar demographic, social, or economic contexts as the Philippines.

## 6. Summary

This article provides an overview of the participant profiles from the HAPPY Senior Citizens study and details the methodologies used to assess the health and nutrition status of community-dwelling older Filipino adults. It serves as a precursor to a series of future publications that will delve deeper into key variables and health outcomes, such as sarcopenia and frailty, and their relationships with body composition, health and nutrition status, functional capacity, and quality of life, among others. The data generated from this study will offer valuable, science-based insights to deepen our understanding of the physiological, clinical, and environmental factors which influence healthy aging in the Philippines.

## Funding

This study is the first part of the HAPPY Senior Citizens R&D programme titled “Relationship of Body Composition to the Functional Capacity and Quality of Life of Older Filipinos in Selected Provinces in the Philippines” funded by the Department of Science and Technology through the Grants-in-Aid program.

## Use of AI tools declaration

The authors declare they have not used Artificial Intelligence (AI) tools in the creation of this article.


