# Supplementary material for: An overview of the healthy aging program for PinoY (HAPPY) senior citizens research: A cross-sectional study among community-dwelling older Filipinos
Source: AIMS Public Health. 2025 May 9;12(2):536–56. doi: 10.3934/publichealth.2025029 (PMC12277775; doi:10.3934/publichealth.2025029)
Supplement: Supplementary file 1 [file publichealth-12-02-029-s001.pdf]

AIMS Public Health

AIMS Public Health, 12(2): 536–556.
DOI: 10.3934/publichealth.2025029
Received: 18 December 2024
Revised: 05 March 2025
Accepted: 17 April 2025
Published: 09 May 2025

https://www.aimspress.com/journal/aimsph

Research article

An overview of the healthy aging program for PinoY (HAPPY) senior citizens research: A cross-sectional study among community-dwelling older Filipinos

Robby Carlo A. Tan[1,2,*], Kyler Kenn M. Castilla[1], Angely P. Garcia[3], Kristine D. Macatangay[1], Shelley Ann F. de la Vega[3], Michael E. Serafico[1], Marco Mensink[2] and Lisette de Groot[2]

[1] Department of Science and Technology, Food and Nutrition Research Institute, Taguig City, Metro Manila, Philippines
[2] Division of Human Nutrition & Health, Wageningen University & Research, Wageningen, the Netherlands
[3] Institute on Aging, National Institutes of Health, University of the Philippines Manila, Manila City, Metro Manila, Philippines

* **Correspondence:** Email: robbycarlo.tan@wur.nl; Tel: +639173077240.

Supplementary File 1. Detailed methodology of the variables collected.

2.4. Variables collected

2.4.1. Sociodemographic information

A researcher-administered questionnaire was performed to gather data on age, birthplace, sex, civil status, household size, length of stay in the household, educational attainment, financial resources, occupational history, toilet type, source of drinking water, mode of food preparation, and health care arrangements.

2.4.2. Anthropometry and body composition

Body circumferences were measured using a retractable, non-stretchable tape (Seca® 201, Hamburg, DE). Waist circumference was measured at the midpoint between the lowest rib and the iliac crest. Hip and calf circumferences were measured at the point of largest circumference over the buttocks and calf, respectively [27,28]. Mid-upper arm circumference was taken at the midpoint between the acromion and the olecranon process, with the arm hanging loosely by the side [18]. Circumferences were taken such that the tape was firmly wrapped around the point of measurement, yet not squeezing the skin.

Knee height was obtained using a large caliper (Seca® 207, Hamburg, DE) in a sitting position and the leg bent at a 90° angle. The adjustable blade of the caliper is placed on the anterior of the left thigh, and the fixed blade is under the left foot's heel with the caliper shaft parallel to the fibula [29]. Measurements were taken twice. A third measurement was taken if prior measurements had more than 0.5 cm difference.

Demi-span was measured in a standing erect position with one arm stretched parallel to the shoulder. Distance from the midpoint of the sternal notch to the web between the middle and ring fingers was measured. The same procedure was repeated for the other arm [29].

Height was measured using a portable stadiometer (Seca® 213, Hamburg, DE) without any footwear and headwear. The back of the head, shoulder, buttocks, and heels fully touched the surface of the stadiometer in a Frankfurt plane position [18]. Two consecutive measurements were performed.

Weight and body composition were collected using a multi-frequency segmental body impedance analyzer (BIA) (Tanita® MC-780MA, Tokyo, JP) following the manufacturer's protocol. The BIA equipment was set on flat, solid, and sturdy ground. Study participants were asked to wear minimal clothing without any metal accessories while in a fasted state or with minimal food and liquid intake prior to measurement. They were asked to stand barefoot on the platform electrodes, arms not touching the sides of the body and hands gripping the left and right handles. After every measurement, information on fat mass, fat-free mass (total and segmental), body water, impedance, and phase angle were saved in the database using Tanita GMON software. Participants with pacemakers and/or metal implants were not assessed for body composition. Nutritional status by Body Mass Index (BMI) was determined using the WHO BMI classification [18].

2.4.3. Biological samples

Participants were informed to undergo 10-12 hours of fasting and were requested not to drink alcoholic beverages nor smoke 24 hours prior to the day of collection. A trained, licensed medical technologist extracted 8 cc to 10 cc of blood via venipuncture. Blood centrifuged to separate plasma, serum, and red blood cells before storing them in tubes. All samples were stored in chilled (-10oC to 0oC) containers or in ice chests for transport to the laboratory. Urine specimens were collected early in the morning and stored in sample containers for urinary iodine analysis. The handling and transport of blood and urine specimens was performed based on the standardized procedures of the DOST-FNRI Service Laboratory Group (SLG). Blood analyses (hemoglobin, lipid profile, c-reactive protein, and

glycosylated hemoglobin) and Urinary Iodine Excretion analysis were performed at the DOST-FNRI SLG following standard laboratory protocol. Cut-off values used were aligned with the NNS [18].

With the growing interest in microbiome studies in aging, a pea-size stool sample was collected from a randomly selected subsample of 20% of the total participant in each city. Stool samples were stored in –20oC freezer. In addition, a spare 2 cc of blood samples were stored in an ultra-low freezer for future research interest in the field of metabolomic and nutrigenomic studies.

2.4.4. Dietary intake

A trained registered nutritionist-dietitian administered the 110- item food frequency questionnaire with the past three months as the reference period to determine the habitual intake. Food items were identified based on the commonly consumed food groups from the NNS database [30,31]. Each item has a frequency-based 12-point ordinal scale with responses ranging from “Never” to “3x per day”. Portion size was estimated using food images and standard household measuring tools, i.e., cups and spoons. Following a standardized procedure, the estimated daily intake of food was calculated by multiplying the frequency of food consumption of each food item by the portion size. The result is converted into daily macronutrient consumption (kcal, protein, carbohydrate, and fats) and is computed using the 2019 Philippine Food Composition Tables [31].

2.4.5. Physical activity level and lifestyle

The World Health Organization (WHO) Global Physical Activity Questionnaire was used to capture information on physical activity. The 16-item questionnaire collects data on three domains: at work, during travel to and from places, recreational activities, and one sedentary behavior (sitting). The frequency per week (days) and duration (minutes) spent performing different activities by intensity (moderate and vigorous) and average time spent sitting in a day were asked. Physical activity can be described in Metabolic Equivalent of Task (MET)-minutes per week and/or as a categorical classification based on the WHO recommendations on physical activity for health [32].

2.4.6. Functional capacity and physical performance

Functional capacity through grip strength was assessed using hand-held dynamometry (Jamar®, IL, US). Participants were asked to remove any wrist/hand accessories as they sat comfortably with back support but without armrests. Shoulders were adducted and neutrally rotated, elbow flexed at 90 degrees, forearm neutral, and wrist positioned in slight extension. Three (3) hand grip measurements were recorded for each hand, alternating sides. The max of three readings for each hand in all trials was used as the final grip strength measurement [33].

The Timed Up-and-Go (TUG) test and the Short Physical Performance Battery (SPPB) test were used to assess physical performance. This measures functional mobility and overall lower extremity function among older people, respectively [34]. To perform the TUG test, participants were timed to sit on a standard-height chair, walk towards a marker 3-ft (0.914 m) away from the chair, then return

to the starting point as fast and safely as possible. Two trials were completed, and the average time was computed for analysis. If the score was ≥13.5 seconds, it was at risk for falling [35].

The SPPB has three sub-components: Balance, Gait Speed, and Lower Leg Strength. Under the balance subcomponent, participants maintained three different stances (side-by-side stance, semi-tandem stance, and tandem stance) for a maximum of 10 seconds each. Participants were assessed on the duration they could maintain each stance. Participants were timed to walk along a 4-meter pathway at a normal speed twice to measure gait speed. The shorter time between the two trials was utilized as the final time. Participants were timed to complete sit-to-stand movements repeated five times to measure lower leg strength. The SPPB is evaluated under a scoring system and each subcomponent has a different criterion to acquire a certain score. Each subcomponent has a maximum of 4 points, making 12 points for the entire SPPB test.

For the 6-minute walk test, the completion time of participants walking a 20-meter pathway twenty times in a comfortable manner was assessed. Those who were unable to complete the course and who completed the test for more than 6 minutes were evaluated to have reduced cardiovascular endurance. The 6-Minute Walk Test was performed to measure aerobic capacity and endurance.

2.4.7. Sarcopenia and frailty screening

The SARC-F questionnaire is a screening tool that screens patients for self-reported signs suggestive of sarcopenia [36]. It is composed of five questions which include deficiencies in the following: 1) strength; 2) assistance in walking; 3) rise from a chair; 4) climb stairs; and 5) falls. Each self-reported parameter receives a minimum and maximum score of 0 and 2, respectively, with the greatest maximum SARC-F score being 10.

The Frailty Index was assessed using Fried's frailty criteria, specifically shrinking, physical endurance/energy, low physical activity, weakness, and slow walking speed. Each criterion has a frailty cut point which determines the overall frailty status of the participant. Based on the scores, the participants can be divided into three stages: non-frail or robust (score 0), pre-frail (score 1–2), and frail (score 3–5) [37].

Shrinking was identified with self-reported unintentional weight loss ≥10 lbs (4.5 kg) the previous year. However, Body Mass Index (BMI) can also be considered as the frailty cut point. If the participant reported unintentional weight loss or if BMI was less than 18.5kg/m2, frailty was present for this criterion.

Physical endurance was identified by two questions from the Center for Epidemiologic Studies Depression scale: 1) "Do you feel full of energy?" and 2) "During the last four (4) weeks, how often have you rested in bed during the day?". Response options for the second question were "every day", "every week", "once", or "not at all". The frailty was present for this criterion if the participants answered "No" to the first question and if the response to the second question was either "every day" or "every week."

Low physical activity was identified through the frequency of engaging in mildly energetic, moderately energetic, and very energetic physical activity. Individuals may respond with "≥3 times per week'', "1–2 times per week", "1–3 times per month", or "hardly ever/never." Frailty was present

for this criterion if participants responded "hardly ever/never" for performing moderately energetic physical activity.

Weakness, on the other hand, was determined through average measurement of hand grip strength based on BMI, age, and sex of the participant. Frailty was present for this criterion if participants did not reach the average hand grip strength set for their age, sex, and BMI.

Slow walking speed was identified based on the Timed Up-and-Go Test (TUG) average time.

2.4.8. Comprehensive geriatric assessment

The comprehensive geriatric assessment is a multidimensional diagnostic process which aims to derive a holistic approach to maximizing the overall health of the aging population. The Filipino version of the form [38], a questionnaire-based assessment used to assess the older person, used in this research was translated and validated by the Institute on Aging of the National Institutes of Health, University of the Philippines Manila, Philippine College of Geriatric Medicine, and Department of Health.

The components of the CGA was used in this study include 1) Problems with senses - oral, eyesight, hearing, 2) Sleeping patterns/problems, 3) Fall history and walking problems, 4) Medical history - illnesses, surgery, medications, 5) Nutrition – supplements, 6) Immunization history, 7) Lifestyle and self-care - vices, physical activity, 8) Review of systems, and 9) Physical and mental health status.

In addition to these components, basic activities of daily living (ADL), including bathing, dressing, toileting, maintaining continence, feeding, and transferring; and instrumental activities of daily living (IADL), including using the telephone, shopping, food preparation, housekeeping, laundry, transportation, taking medicine and managing money were assessed. Scores are based on the capacity of the participants to independently perform the activities to being unable to perform the activities. Furthermore, blood pressure was also taken using a non-mercurial sphygmomanometer (UM-102) placed around the arm over the brachial artery while the participant was seated comfortably. Measurements were taken thrice with at least a minute of rest between measurements. The average of three readings was taken as the final measurement [18].

The research team underwent training supervised by the University of the Philippines National Institutes of Health (UP NIH) - Institute on Aging to be equipped in assisting during the conduct of CGA by the geriatrician or physician. After the interview, the CGA-trained geriatrician or physician reviewed the responses before proceeding to the physical and mental examination and diagnosis.

2.4.9. Quality of life

Perception of their overall quality of life was assessed using the World Health Organization Quality of Life Brief Version (WHOQOL-BREF) which has been culturally validated for Filipino older adults [39,40]. The 26-item questionnaire covers four domains of life including physical health, psychological, social relationships, and environment. Each item is scored through a 5-point ordinal scale dependent on the participant's degree of perceived capability, frequency, or contentment on

specific facets of their life within the last two weeks. An additional question on perceived challenges during the time of COVID-19 was added to gather their insights and experiences.

2.4.10. Geographical information system

The locations of households and landmarks of each barangay were recorded using Global Positioning System (GPS) from smartphones (Save Location app). These GPS points were verified and mapped in Google Earth together with the environmental data. Additionally, environmental data related to neighborhood greenness, urbanization, population, food proximity, and topography were also identified. Using geospatial software, the nutrition and health outcomes can be mapped at neighborhood levels to provide spatial inform.

AIMS Press

© 2025 the Author(s), licensee AIMS Press. This is an open access article distributed under the terms of the Creative Commons Attribution License (https://creativecommons.org/licenses/by/4.0)
